# Transfer learning for metamaterial design and simulation

**DOI:** 10.1515/nanoph-2023-0691

**Published:** 2024-03-22

**Authors:** Rixi Peng, Simiao Ren, Jordan Malof, Willie J. Padilla

**Affiliations:** Computer Science, University of Montana, Missoula, MT, USA; Electrical and Computer Engineering, Duke University, Durham, NC, USA

**Keywords:** deep learning, transfer learning, metamaterials, scattering, metasurfaces

## Abstract

We demonstrate transfer learning as a tool to improve the efficacy of training deep learning models based on residual neural networks (ResNets). Specifically, we examine its use for study of multi-scale electrically large metasurface arrays under open boundary conditions in electromagnetic metamaterials. Our aim is to assess the efficiency of transfer learning across a range of problem domains that vary in their resemblance to the original base problem for which the ResNet model was initially trained. We use a quasi-analytical discrete dipole approximation (DDA) method to simulate electrically large metasurface arrays to obtain ground truth data for training and testing of our deep neural network. Our approach can save significant time for examining novel metasurface designs by harnessing the power of transfer learning, as it effectively mitigates the pervasive data bottleneck issue commonly encountered in deep learning. We demonstrate that for the best case when the transfer task is sufficiently similar to the target task, a new task can be effectively trained using only a few data points yet still achieve a test mean absolute relative error of 3 % with a pre-trained neural network, realizing data reduction by a factor of 1000.

## 1 Introduction

The fields of photonics, plasmonics, metamaterials, and metasurfaces, along with other artificial electromagnetic material (AEM) systems, have witnessed remarkable advancements facilitated by the maturation of deep learning (DL) techniques [1], [2], [3]. Numerous studies have highlighted the benefits of employing data-driven approaches, such as accelerated modeling and inverse design of complex systems [4], [5], [6], [7], [8]. Traditionally, computational electromagnetic solvers (CEMS) such as finite difference time domain (FDTD) and the finite element method (FEM) have been extensively used in AEM research, effectively solving Maxwell’s equations and enabling accurate design for both applied and basic research pursuits [9], [10], [11], [12]. However, CEMS operate as grid solvers, requiring complete re-simulation of the entire problem even for minor changes in geometry. While this may not pose significant challenges for simple geometric arrangements or single unit-cell periodic systems, it becomes burdensome for electrically large arrays, often taking hours or even days for simulation [13], [14], [15]. In contrast, deep learning offers a compelling alternative by training neural networks on a *relatively* small set of ground truth CEMS simulations to create surrogate (proxy) models for a specific metasurface system [16]. When using deep learning, one typically creates a dataset of the form $D={\left(g_{i},s_{i}\right)}_{i=1}^{N}$ using CEMS, where *g*_*i*_ is a vector quantifying the geometric structure of the AEM (e.g., the width of a wire or the periodicity of a unit-cell), and *s*_*i*_ refers to the corresponding scattering properties of interest. It is then assumed that there exists some function *f* relating the geometric structure of the AEM to its scattering properties, i.e. *s* = *f*(*g*). Our aim is then to approximate *f* with a deep neural network (DNN), denoted $\hat{f}\left(g;\Theta \right)$ , where Θ refers to all the parameters of the DNN model. We then utilize the example pairs in *D* to infer a setting of Θ such that $\hat{f}\left(g;\Theta \right)$ produces highly accurate predictions for all of the data in *D*. This process of adjusting *θ* to achieve good prediction accuracy is called *training*, and *D* is referred to as the *training dataset*. Once trained, the model $\hat{f}\left(g;\Theta \right)$ can be used to predict *s* for new settings of *g* that were not present in *D*, and do so substantially faster than the CEMS that was used to create *D*. Often $\hat{f}$ can make predictions several orders of magnitude faster than its associated CEMS (e.g., nearly 10^6^ times faster [5]), at the cost of imperfect prediction accuracy; for this reason $\hat{f}$ is often referred to as a surrogate model of *f*.

Deep learning – and DNNs in particular – have found success as surrogate models for a wide variety of problems, enabling the rapid exploration of the scattering properties of AEMs of unprecedented complexity. DNNs have also displayed considerable potential in tackling inverse problems within AEM studies, wherein the goal is to infer the geometric structure needed to obtain some designed scattering response (i.e., find *g* given some setting of *s*) [1], [8]. However, generating the dataset to train the DNNs can be time-consuming and even impracticable in computational, and especially experimental, scenarios depending on the problem’s complexity and the design space’s size. It is important to note that the required dataset size for a specific level of accuracy in deep learning is unknown *a priori*, potentially introducing significant risk into its use in resource-constrained settings. Additionally, this data bottleneck issue persists even when deploying deep learning to solve new – but highly similar – problems, as this requires the procurement of a completely new training dataset. For example, two problems may have identical geometric parameterizations but need to be solved in different frequency ranges (i.e., same *g* but different *s*), or they may share the same spectral range but have distinct geometric parameterizations (i.e., same *s* but different *g*). In both cases, data generation must start anew, as a well-trained network’s accuracy is limited to the specific problem it was trained on. However, when two problems are related, it may be possible to leverage patterns observed in one problem’s data – referred to as the *source problem* – to reduce the amount of new training data needed to achieve satisfying accuracy on the second distinct problem, called the *target problem*. This general concept, known as *transfer learning* in machine learning literature [17], [18], has proven highly effective in mitigating the data bottleneck problem across various applications, such as image processing [19], [20] and natural language processing [21].

**Figure 1:**
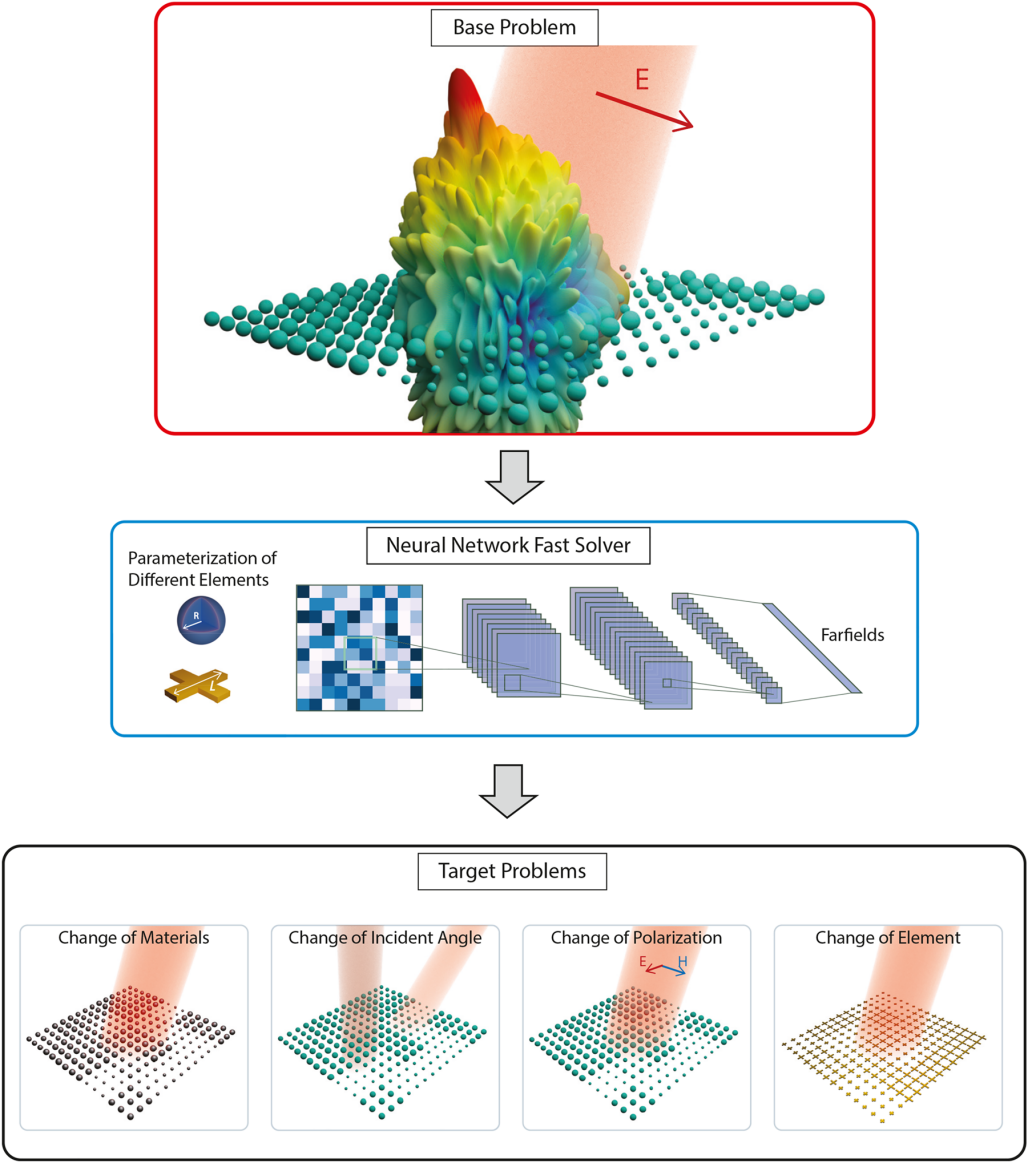
Transfer learning from the source problem and the corresponding neural network to multiple target problems. Top: Scattering from an array of dielectric spheres. The incident field (red) gives rise to the farfield scattering pattern plotted as magnitude (colormap) on a 3D surface and polar radius. Middle: Neural networks as surrogate solvers. A neural network takes the geometry parameters of elements in an array, formed as a numeric matrix, and outputs the farfield patterns. Bottom: Schematics of different problem changes (target problems) from the top one (source problem).

DNNs are typically trained using mini-batch gradient descent, which is an iterative process where the model parameters, Θ, are repeatedly updated using small subsets of the training data. The model parameter settings at the outset of training can have a significant impact on the final performance of the model. In conventional training the model parameters are initialized to random values, using some predefined sampling distribution, such as Xavier or Kaiming initialization [22]. The approach for transfer learning, however, is a two-stage process. The first step involves training a DNN on the *source task*, and the second stage is to use the resultant accurate model parameters as an initialization for the training process on the *target task*. In this approach, the model is said to be *pre-trained* on the source task, and then *fine-tuned* on the target task. The transfer learning method has been found to be highly effective in a wide variety of application domains, often dramatically improving model performance on the target tasks, see Figure 1.

Recently, several attempts have been made to apply transfer learning methods to AEM problems [23], [24], [25], [26]. These studies use a common input parameterization space and treat a metamaterial element or array as an image. For example, in [23], [24], transfer learning is applied to different discrete image representations of a two-dimensional shape consisting of a single metamaterial element. Another method examined an array-level problem in an inverse approach, solving for intermediate physical quantities, such as the phase map, for the target scattering properties, such as farfield patterns [25], [26]. However, our study distinguishes itself from other works by accepting a much more general input, which permits the study of drastically different external physical conditions. In our work, each entry in the input array represents an abstract geometric parameter of the element, such as the radius or the length, resulting in a simple and scalable representation of the geometric space. We solve the direct problem to obtain the scattering properties resulting from a diverse set of geometries. Our work presents a more versatile transfer learning application valid for a much broader range of AEM problems than the aforementioned works.

Despite the effectiveness of transfer learning shown in some recent works, it is still limited to relatively similar problems with a shared input space. In addition, pre-training is seldom used in AEM applications for several reasons. First, the geometric parameterizations of AEM problems vary widely, meaning that the definition and dimensionality of the input space, *g*, can differ significantly. This makes it difficult to apply transfer learning when the dimensionality of the source and target problems is different. Second, transfer learning is most effective when the meaning of the input parameters is similar across the source and target problems. This condition is rarely satisfied across AEM problems that have been studied with deep learning. In [27], stacks of both spherical and planar layers were studied where the definition of the thickness was different between the two problems, but still related via a coordinate transform. However, for more significant changes in an input geometrical parameter the settings of *g* can refer to fundamentally different geometric structures of the AEM, making pre-training difficult or less effective. Finally, it is not currently common practice for authors in the AEM community to share their CEMS datasets or pre-trained model parameters, which makes it difficult for other researchers to fine-tune these models on new AEM tasks. In this work, we aim to explore the use of transfer learning on a much wider range of AEM problem types where physical conditions change and the input space does not have the same meaning for different problems.

### 1.1 Contributions of this work

In this work we demonstrate the tremendous potential benefits of transfer learning for AEM tasks, motivating its broader adoption in the AEM community. To study the benefits of transfer learning, we use it to develop surrogate DNN-based models for the multi-scale problem of scattering from electrically large random metasurface arrays – a problem commonly encountered in applications such as metamaterial lens design [28], [29], disordered metasurfaces [30], [31], and Huygens metasurfaces [32]. We begin by generating a large number of simulations for a source task, and then explore several different target tasks with varying problem features – the base material, geometric shape, incident angle, and polarization of the excitation. We evaluate the accuracy of DNN-based surrogate models on each target task as a function of the quantity of data used to train the model, and perform a direct quantitative comparison to the accuracy of source tasks with and without transfer learning. Our results indicate that, using transfer learning, we can often dramatically reduce the quantity of training data that is needed to achieve a desired prediction accuracy for new problems, e.g., often over an 80 % reduction, and as high as 90 %. Crucially, we also find that transfer learning is never detrimental over our diverse target problem settings, suggesting that there is little risk in utilizing it.

To enable the benefits of transfer learning, we propose a geometric parameterization that makes transfer learning straightforward across all of the proposed tasks, and allows us to leverage existing successful DNN-based models from the computer vision literature. We therefore represent the geometric structure of the AEMs as a 2D array – similar to an image – where each entry in the array corresponds to a geometric parameter of the unit-cell, as illustrated in Figure 1. This structure allows us to adopt convolutional DNN model architectures from the computer vision community, which can take advantage of the spatial structure of the underlying metasurface when making predictions. This parameterization also means that little or no modification is required to utilize pre-trained model parameters from the source task, making it easy to use pre-trained weights for each target task.

This paper is organized as follows. First, we present the analysis of using a residual neural network structure to solve a base metamaterial array scattering problem, which serves as the source problem from which we transfer knowledge. Second, we briefly introduce the discrete dipole approximation (DDA) method for efficient data generation. Next, we explore five target tasks involving various condition changes, including element material, shape, and excitation. Finally, we perform a quantitative analysis on the effectiveness of transfer learning in terms of error reduction as a function of dataset size, as well as data reduction for given levels of accuracy. For each target task we also make a comparison between the transfer learning approach and for the case where we use random initialization and do not use any transfer knowledge.

## 2 Methods

### 2.1 Array scattering problem settings

We aim to solve the scattering problem for different random arrays under similar, yet distinct, physical conditions. Specifically, one class of problems, or equivalently one task, examines scattering from a random metasurface array consisting of elements with distinct morphology but with fixed periodicity and excitation fields. Another problem class involves array scattering from the base problem, but with different excitation fields. In each task instance, the input variables are the geometric parameters of different random arrays, denoted as *g*, and the output variables are the far-field patterns, i.e., the electromagnetic responses of the array, denoted as *s*.

### 2.2 Deep convolutional neural network

The scattering patterns from the metamaterial array are determined by the specific spatial arrangement of single elements in the array subject to different excitations and other environmental conditions. Therefore to make our method as general as possible, i.e. transferable to as many different types of metamaterials and physical scenarios as possible, we represent the geometry space *g* as a numeric matrix. This numeric matrix *g* is of the size *C* × *N* × *M*, where *C* represents the total number of tunable geometric parameters for each single element, e.g. some critical length or radius, and *N*, *M* are the 2D array sizes. Therefore, large arrays of metamaterial elements can be characterized in a compact and scalable fashion even if the elements are three-dimensional with fine features. The use of a CNN also permits preservation of the spatial relationship between elements in the input numeric matrix, thereby allowing the convolutional filters to learn element interactions.

While the geometric layout can be readily represented using matrices, the random array far-field radiation pattern is generally a function of 3D spherical angles, as depicted in Figure 1. For simplicity, and without loss of generality, we consider only one representative slice in the 3D radiation pattern along a constant forward scattered azimuthal angle. The output far-field patterns |*E*| are sampled along the forward scattering direction with varying elevation (polar) angles from 0 (North Pole) to 180 (South Pole) degrees, represented by a 181 × 1 positive number vector, so that $s\in R^{+,181}$ .

Due to the large magnitude variations of the far-field patterns, we formulated a mixed linear-log loss, which we found to be more effective, in comparison to the traditional MSE loss (applied in either the linear or log scale.) We define our training loss as:

(1) $$Loss=\frac{1}{N}\underset{i}{\sum }\left[{\left|s_{i}-f\left(g_{i}\right)\right|}^{2}+\alpha {\left|h\left(s_{i}\right)-h\left(f\left(g_{i}\right)\right)\right|}^{2}\right],$$

where *h*(*x*) = ln(1 + *x*). The log-scale term is used to decrease the loss for the scattering pattern expressed in a dB scale. We found for the hyperparameter *α* that *α* = 5 is a suitable choice for the far-field scattering problem in this paper, which balances the loss between the linear and logarithm scales. For the test set we use the mean absolute relative error (MARE), defined as:

(2) $$MARE=\frac{1}{N}\underset{i}{\sum }\frac{\left|s_{i}-f\left(g_{i}\right)\right|}{\left|s_{i}\right|}.$$

This metric ensures that the reported loss is independent of the absolute magnitude level of the far-field signal, making it suitable for comparisons across different problems.

CNNs can be constructed with different architectures, which refer to the order in which data is processed (e.g., its connectivity) and the specific processing that takes place. In this work, we use a residual convolutional neural network (ResNet), which has been found highly effective for computer vision problems [33] by exploiting the local information between close neighbors – similar to the input data we consider here. Using a 2-dimensional matrix encoding of our material design, we can leverage state-of-the-art CNN architectures from the computer vision literature. Furthermore, we hypothesize that the 2-D ResNet convolution operations resemble the far-field inference procedure employed for Green’s functions, and therefore, may be well-suited for modeling far-field radiation patterns. Our ResNet consists of one base convolution layer, followed by six residual blocks with (64, 64, 128, 128, 256, 256) convolution channels. Each residual block in the ResNet architecture consists of two identically sized convolutional layers connected by a skip connection. Each convolutional (Conv) layer is followed by a batch normalization (BN) layer and a ReLU activation. The base convolution kernel has a size of 7 × 7, and the remaining convolutional layers have a kernel size of 3 × 3. These residual blocks are followed by one max pooling layer of 2 × 2, and one fully-connected layer to yield the 181 × 1 vector output. If sufficient training data are collected and used to train a CNN model such that $s=\hat{f}\left(g;\Theta \right)$ predicts accurate responses, the $\hat{f}$ surrogate network model can be generalized to solve the scattering problem of any new random array within the training domain, which is not in the training dataset, without expensive full-wave computation. In this context, the CNN model serves as a fast solver for the random array scattering problem. Note that this trained CNN surrogate model $\hat{f}$ is tied to the problem with all the physics conditions fixed, (the element material, shape, and incident fields), and all implicitly embedded in the network parameters.

### 2.3 Transfer learning using fine-tuning

As mentioned, the trained CNN model is accurate only for the dataset on which it was trained, and therefore cannot be used directly to solve a new task. An entirely new dataset *D*_*tgt*_ for the target problem needs to be generated, and a new CNN must be trained using *D*_*tgt*_. However, if the new task shares some common underlying physics, transfer learning can leverage the information incorporated in the trained CNN for the new task. We begin with an initial modeling problem, termed the *source problem*, for which a large training dataset exists, denoted *D*_*src*_. We then train a ResNet model on *D*_*src*_, resulting in a trained CNN with weights *θ*_*src*_ that achieve highly accurate predictions for (*g*, *s*) ∈ *D*_*src*_. In the *target problem*, denoted *D*_*tgt*_, we vary some aspects of the experimental scenario of the base problem, such as the geometry or excitation. In the traditional approach, we would need to collect a large quantity of data again to obtain accurate model parameters for *D*_*tgt*_; however, we hypothesize that initializing the gradient descent training procedure with the model parameters *θ*_*src*_ will lead to improved performance compared to randomly initializing the model parameters – the procedure typically used when training CNNs for a new task.

### 2.4 Discrete dipole approximation method for scattering

To generate the dataset in a practically realizable time, a semi-analytic method called the discrete dipole approximation (DDA) is used as an alternative to commercial full-wave simulation software. A finite-sized metasurface array may consist of dielectric or metallic elements and may be of large electrical size. It is computationally expensive or even impossible to simulate the entire array depending on the electrical length and desired wavelength range, which specifies the grid size and therefore the total problem size. The local slowly-varying approximation (SVA) is usually applied to these types of array problems by solving for the electromagnetic response of a single metamaterial element with periodic boundary conditions, thereby modeling an infinite identical array [34]. The local periodic response is then used to approximate the actual response for the element when embedded in a random array. The SVA is valid if the response fields from element to element are smooth, and the change in the coupling between elements, due to the random arrangement, is negligible. However, this method may result in poor accuracy since the local response of one element can be drastically different from the periodic response of the same element with different neighboring elements. The problem we explore here cannot be studied with SVA and therefore we use the polarizability retrieval method together with the discrete dipole approximation method to extract the response of the elements embedded in a random array. The DDA method, along with the retrieval method, enables accurate and fast large-area metasurface simulation.

Many sub-wavelength metasurfaces can be accurately approximated by an electric dipole $\bar{p}$ and a magnetic dipole $\bar{m}$ . The polarizability tensor $\bar{\bar{\alpha }}$ relates $\bar{p},\bar{m}$ with the local field *E*, *H* by

(3) $$\bar{\bar{p}}=\left[\begin{array}{cc} {\bar{\bar{\alpha }}}^{ee} & {\bar{\bar{\alpha }}}^{em}\\ {\bar{\bar{\alpha }}}^{me} & {\bar{\bar{\alpha }}}^{mm} \end{array}\right]\left[\begin{array}{c} E\\ H \end{array}\right].$$

where for example,

(4) $$\bar{\bar{\alpha }}=\left[\begin{array}{ccc} \alpha_{xx} & \alpha_{xy} & \alpha_{xz}\\ \alpha_{yx} & \alpha_{yy} & \alpha_{yz}\\ \alpha_{zx} & \alpha_{zy} & \alpha_{zz} \end{array}\right]$$

**Figure 2:**
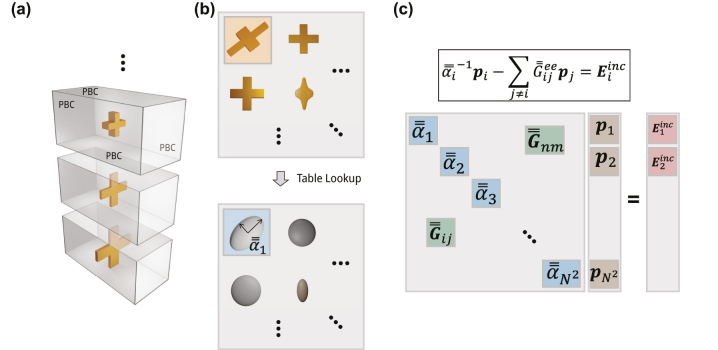
Flowchart of the DDA solver and the neural network proxy. (a) Single element simulations are used to obtain a library of polarizabilities for different elements. (b) Using the polarizability library, an array of elements can be mathematically replaced by a matrix of the corresponding polarizabilities. (c) The scattering from a complex array is reduced to a system of linear equations, where the coefficients are determined by the exact elements and the environment. The right-hand side of the boxed equation is the excitation $E_{i}^{\text{inc}}$ . Any problem change can be directly modified in this system of linear equations.

The polarizability tensor $\bar{\bar{\alpha }}$ can accurately describe both the material parameters and geometric features of a metamaterial element in a periodic environment. Therefore we may build up a library of $\bar{\bar{\alpha }}$ ’s for a series of desired metamaterial unit-cells which we intend to use for a given problem, as depicted in Figure 2. The polarizability retrieval method entails performing several scattering parameters (S-parameters) simulations to retrieve $\bar{\bar{\alpha }}$ [35]. A 4 × 4 S-parameter matrix, corresponding to 2 ports each with 2 orthogonal polarization states (*h*,*v*), of a single metamaterial element with periodic boundary conditions is found through full wave simulations. Note that the full wave simulation is only used in this step for each given single element. The inverse retrieval operator *F* is then applied on the S-parameters to get the 4 × 4 in-plane sub-tensor of the full 6 × 6 polarizability tensor, given by ${\bar{\bar{\alpha }}}_{xy}=F{\left[S\right]}_{4\times 4}$ . To retrieve the full 6 × 6 polarizability tensor, one or two additional simulations may be required where the element is rotated about a principle axis. This technique is applied for every metamaterial unit-cell geometry and frequency of interest in advance to generate the {geometry; polarizability} library which can then be configured in any arrangement to approximate an array of actual metamaterial elements.

With a polarizability tensor determined for every desired metamaterial element, the random metasurface array scattering problem is then reduced to solving a linearized integral equation system which, for an arbitrary *j*th metamaterial element in the array, is described by,

(5) $$\left[\begin{array}{c} {\bar{p}}_{j}\\ {\bar{m}}_{j} \end{array}\right]={\bar{\bar{\alpha }}}_{j}\left[\begin{array}{c} E_{0}\\ H_{0} \end{array}\right]+\underset{i\neq j}{\sum }{\bar{\bar{\alpha }}}_{i}\left[\begin{array}{cc} {\bar{\bar{G}}}_{ij}^{ep} & {\bar{\bar{G}}}_{ij}^{em}\\ {\bar{\bar{G}}}_{ij}^{hp} & {\bar{\bar{G}}}_{ij}^{hm} \end{array}\right]\left[\begin{array}{c} {\bar{p}}_{i}\\ {\bar{m}}_{i} \end{array}\right],$$

where *E*_0_, *H*_0_ are the incident electric and magnetic fields at the *j*th element location, and $\bar{\bar{G}}$ is the Green’s function for the environment. The superscripts on the Green’s function stand for electric field (*e*), magnetic field (*h*), electric dipole (*p*), and magnetic dipole (*m*). The above scattering equation holds for every element in the metamaterial array, leading to a linear equation system. Then Eq. (5) can be solved by multiple standard methods, for instance, generalized minimal residual method (GMRES) to obtain ${\bar{p}}_{j},{\bar{m}}_{j}$ for every element in the array.

The far-field pattern is the coherent summation of the radiated fields from all the electric and magnetic dipoles with the far-field approximation, i.e. $E\left(\theta ,\phi \right)=\sum_{i}\left(E_{i}^{p}+E_{i}^{m}\right)$ . For an individual electric dipole the free space far-field radiation is given by,

(6) $$E_{i}^{p}\left(\theta ,\phi \right)=\frac{k^{2}}{4\pi \epsilon_{0}}\left(\hat{n}\times {\bar{p}}_{i}\times \hat{n}\right)\frac{e^{-jkr}}{r}$$

and for a magnetic dipole by,

(7) $$E_{i}^{m}\left(\theta ,\phi \right)=-\frac{Z_{0}k^{2}}{4\pi }\left(\hat{n}\times {\bar{m}}_{i}\right)\frac{e^{-jkr}}{r}$$

where $Z_{0}=\sqrt{\mu_{0}/\epsilon_{0}}$ , *ϵ*_0_ and *μ*_0_ are the permittivity and permeability of free-space, respectively, $\hat{n}$ is the surface normal of the array, and *k* is the magnitude of the free-space wavevector. We note that in the case where dipoles are embedded within a substrate, the radiation is within a dielectric and therefore possesses an analytically solution for the case of planar stratified substrates [36].

## 3 Results

In this section, the training results of using transfer learning for 5 target problems (see Table 1) from one base problem are presented.

**Table 1:** Task list: 1 source problem (base) and 5 target problems.

	Task name	Material	Shape	Incident angle	Polarization
0	Base	Silicon	Sphere	13°	TE
1	0° incident angle	Silicon	Sphere	0°	TE
2	30° incident angle	Silicon	Sphere	30°	TE
3	TM polarization	Silicon	Sphere	13°	TM
4	PEC spheres	PEC	Sphere	13°	TE
5	Al crosses	Aluminum	Cross	13°	TE

### 3.1 Tasks and datasets

All six problems are shown in Table 1, (including the original base problem), and consist of the computed scattered far-fields of a 2D metamaterial array of 50 × 50 randomly chosen elements in free space. As mentioned, each problem differs in either the type of radiation illuminating the array, or in the material and geometry of the array elements. Different instances of these problems, namely the samples in the dataset, are of different individual element sizes with other conditions fixed. The base problem involves calculating the scattered far-field of an array of silicon spheres under a TE polarized plane wave incident at 13° from the surface normal at a wavelength of 11 μm. The dataset for each problem consists of 50,000 training data in total and another 5000 testing data, all generated by the DDA method described here. The geometry parameters for each metamaterial element in the array are randomly drawn from a uniform distribution spanning from 0.45 μm to 1.1 μm, which represents the radius for a sphere array or the half length of an array consisting of metallic cross elements. We have chosen transfer tasks which we expect will have varying similarity to the base problem, and therefore which will yield varying degrees of success. The first three target problems shown in Table 1 each keep constant the metasurface geometry and material, but allow the state of external radiation to vary. In tasks 4 and 5 the base material is changed to PEC and aluminum (Al), respectively, and in task 5 both the material and shape of the metasurface element changes, thus representing a two-factor change from the base problem.

**Figure 3:**
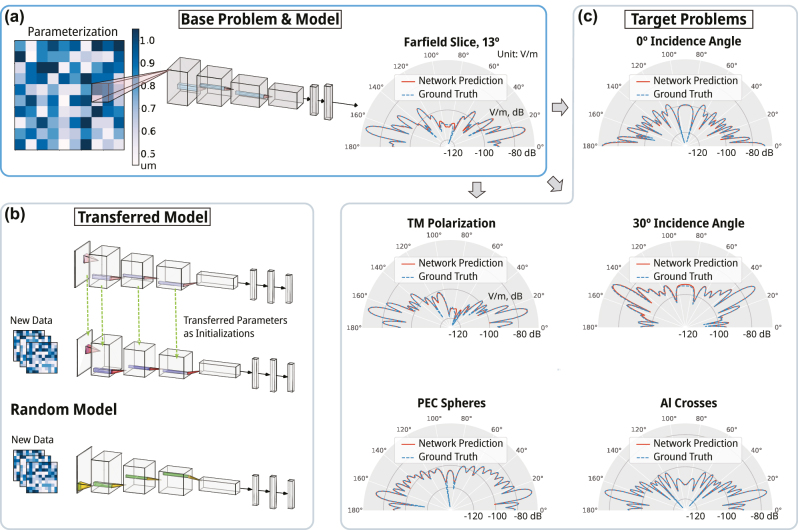
Dataset and random training results: (a) the base problem is the scattering resulting from a 50 × 50 array, consisting of silicon (*ϵ*_*r*_ = 11.3) spheres. The spheres are grouped in a 5 × 5 subarrays with identical radii. The input matrices are shown with the radii ranging from 0.45 µm to 1.05 µm. The forward scattering slice in the farfield pattern is plotted in polar coordinates in dB scale in the original unit *V*/*m* of E field. This slice is at the azimuthal angle of 180° and the elevation angle is ranging from 0 to 180°. Two random sample points from the test dataset are shown for all 6 problems. Ground truths are computed using the DDA method and compared to the output from a trained model using a total of 50,000 data. (b) All the trainable parameters are transferred from the trained base network for the initialization of the neural network for any new problem. Random networks represent the baseline where all the neural network parameters are randomly initialized. (c) The farfield slices of samples from all the five target problems are shown.

In Figure 3(a) we show a schematic of the ResNet used in our study. The geometry of the metasurface array is represented here as a colormap matrix with the colorbar showing the radius of each sphere in the array. The matrix then feeds into the ResNet, which we depict here in AlexNet style [37]. As mentioned, the ResNet is trained on a dataset obtained from our DDA solver and thus the output predicts the far field scattering. To validate the accuracy of our DDA modeling approach we perform a direct comparison to a commercial computational electromagnetic solver. However, CEMS is limited in the maximum array size which can be studied; therefore here we reduce the metasurface array size to 30 × 30. On the right side of Figure 3(a) we show a slice of the far-field radiation taken along *ϕ* = 180° and for polar angles from 0° ≤ *θ* ≤ 180°, where the ground truth is shown as a dashed blue curve and the ResNet prediction is shown as the solid red curve.

Figure 3(b) presents a schematic depicting the transfer learning approach used for our study. The top AlexNet diagram in Figure 3(b) represents the base problem from which we transfer the parameters of the trained ResNet to the new problem under study – middle AlexNet diagram shown. We also compare the transfer learning approach to training a Resnet from scratch, which is shown in the bottom of Figure 3(b). In Figure 3(c) we compare the output of our DDA trained ResNet to computational simulations obtained from CST Microwave Studio for all target problems listed in Table 1. Here the network predictions are shown as solid red curves, while the ground truth simulations are shown as the dashed blue curves. All the neural network predictions are from the best model trained from random intializations using a total of 50,000 data.

### 3.2 Comparison of training from random initialization and transfer learning

To evaluate the effectiveness of transfer learning approach for the aforementioned five target tasks, two training schemes are implemented and compared for each problem. The first approach involves training a neural network with randomly initialized parameters, while the second is the transfer learning approach which involves fine-tuning a neural network initialized with the best model from the base problem – see Table 1. In the experiments conducted, the transferred neural network parameters are obtained from the best training results of the base problem, which used a total of 50,000 data points. Identical datasets of varying sizes are used for both training schemes, ranging from {500} to {1, 000} with a step size of 100, {1, 000} to {10, 000} with a step size of 1000, and {10, 000} to {50, 000} with a step size of 5000. The optimal model for each case is determined by searching over a grid of learning rates ([0.1, 0.01, 0.001, 0.0001]) and regularizers ([0.01, 0.001, 0.0001, 0.00001]). Each experiment is trained five times using the best hyperparameter combination to account for randomness. The resulting best models for both training schemes at different dataset sizes are then evaluated using a test dataset of size 5000. Although the mixed loss shown in Eq. (1) is employed during the training phase, we use a linear MARE loss in the testing phase, and for reporting and visualization purposes. Moreover, the MARE test loss is a normalized measure which enables a comparison among different problem types.

**Figure 4:**
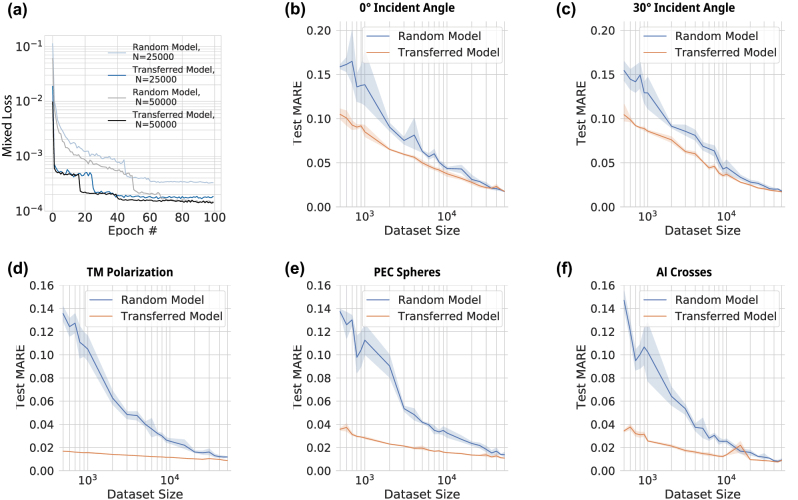
Results of transfer learning and training from scratch for all target problems. (a) The training curves of transferred models and random models using 25,000 and 50,000 new data for the new problem. (b)–(f) The averaged test error of the transferred models and the random models as a function of dataset size. Five repetitive training are used to compute the mean error (solid line) and the 95 % confidence interval (shaded regions).

Figure 4 shows the test MARE of the transfer learning (orange) and random (blue) models as a function of data set size for each target problem. Here the solid curve is the average of the five aforementioned experiments, and the associated shaded area represents the 95 % confidence interval. We find that the transfer learning scheme consistently results in lower test loss for all target problems compared to random initialization. Further, the test MARE difference between transfer learning and random is a maximum for low dataset sizes which gradually diminishes for increasing dataset size. Notably, we do find that the advantage of transfer learning varies among the five target tasks, which can be intuitively observed through the gap between the two loss curves. The benefits of transfer learning are good for the two angle variation cases shown in Figure 4(b) and (c), while the TM polarization case shows the most significant improvement.

### 3.3 Quantitative analysis of the advantage of transfer learning

**Figure 5:**
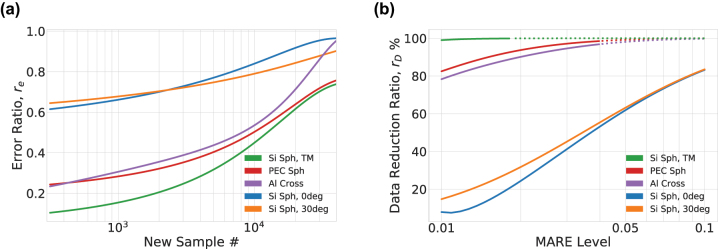
Gain of transfer learning: (a) the ratio of the test loss error of transferred training and that of training from scratch using the same amount of data. (b) The reduction in required dataset size using transfer learning to reach a fixed level of accuracy.

We comprehensively examine the advantages of the transfer learning approach by comparing its performance using two metrics. In the first case we measure the relative reduction in error between the transfer learning case and the random case, assuming a fixed quantity of target-task training data is available – denoted *r*_*e*_. We also examine the relative reduction in training data required in the transfer learning case to achieve a desired level of prediction error, as compared to the random case – denoted *r*_*D*_. To compare the relationship between the dataset size and error, we fit the data from Figure 4 using a third order polynomial to obtain a smooth *e* = *g*(|*D*|), where |*D*| denotes the size of a dataset *D*. Using the fitted functions both *r*_*e*_ and *r*_*D*_ can be calculated and results are shown in Figure 5. The error reduction ratio is a vertical cut of the data presented in Figure 4(b)–(f) and defined as,

(8) $$r_{e}\left(D\right)\equiv \frac{e^{TL}\left(\left|D\right|\right)}{e^{RI}\left(\left|D\right|\right)}$$

where *e* ^*TL*^ is the test MARE using transfer learning at a dataset size of *D*, and *e* ^*RI*^ is the test MARE using random initializations also for *D*. In Figure 5(a) we see that for small data sizes *r*_*e*_ is optimal and, for example at *D* = 300, we find that *r*_*e*_ ≤ 10 % for Tasks 3–5, and *r*_*e*_ ≈ 65 % for Tasks 1 and 2 – see Table 1. For all dataset sizes explored we find that transfer learning always leads to improved accuracy on the test set in comparison to training from random initialization, i.e. *r*_*e*_ is less than unity. However, we find that the advantage of transfer learning gradually diminishes for increasing dataset size, and as we approach *D* ≈ 50, 000 we find *r*_*e*_ → 1.

The data reduction ratio *r*_*D*_ explores the data required for the transfer learning approach to achieve a specific test MARE compared to the data for random initialization to achieve the same test MARE. Therefore *r*_*D*_ are horizontal cuts of the data presented in Figure 4(b)–(f), and defined as,

(9) $$r_{D}\left(e\right)\equiv 1-\frac{D^{TL}\left(e\right)}{D^{RI}\left(e\right)}$$

where *D* ^*TL*^ is the dataset size for transfer learning and *D* ^*RI*^ is the dataset size for random initialization. In Figure 5(b) we plot *r*_*D*_ as a function of new sample simulations. If we take a test MARE of 3 % as a point of comparison, we find that the data can be reduced by over a factor of 2000 compared to random initialization for the TM Task 3 case, whereas we find *r*_*D*_ values ranging from 94 % to approximately 22 % for the PEC Sphere (Task 4) to the 0° (Task 1), respectively. For all Tasks explored we find that a significant reduction in data is achieved for transfer learning compared to random initialization. The exact numbers of dataset sizes or MARE are compared in Table 2 at two critical metrics, i.e. MARE = 0.03 and data of *D* = 1, 000 points. For this comparison, the data of Figure 4(b)–(f) have been interpolated and, as observed from the data presented in Table 2, we find that to achieve a test MARE of 3 %, various tasks require drastically different amounts of data. For example, Task 3 needs only *D* = 3, whereas Tasks (4, 5, 2, 1) required *D* = (776, 924, 13691, 17,353). We note that for TM Task 3 case a third order polynomial fit – rather than the interpolated data – is used for Table 2, as denoted by the asterisk.

**Table 2:** Comparison of transfer learning and random initializations evaluated for critical metrics.

Task	Name	Data required for MARE = 0.03		MARE for *D* = 1000	
		Random initializations	Transfer learning	Random initializations	Transfer learning
3	TM	7441	3^a^	0.101	0.015
4	PEC spheres	12,050	776	0.103	0.029
5	Al crosses	7102	924	0.094	0.028
2	30°	18,113	13,691	0.127	0.086
1	0°	22,158	17,353	0.103	0.029

^a^Value determined using a third order polynomial fit described in the text.

**Figure 6:**
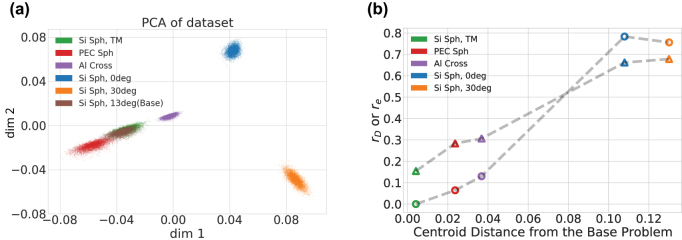
Simularity analysis: (a) PCA representation of the far field scattering data from the six problems. (b) The correlation of the PCA centroid distance from (a) and data reduction ratio (circle symbols) at 3 % MARE and the error ratio (triangle symbols) at *D* = 1000 for each of the target tasks.

The large variance in data required to achieve a certain error level presented in Table 2 hints that some task problems share more or less underlying physics with the base problem. To elucidate this connection, we perform a similarity analysis on the output far field scattering for each of the transfer Task problems. Figure 6(a) shows the results of a principal component analysis (PCA) performed on the farfield scattering patterns of 181 by 1 data points. The analysis utilized a total 60,000 data points, with each Task – including the base Task – contributing 10,000. As evident in the feature-wise basis, there is some overlap between the TM Task 3 (orange) and Base Task 0 (brown) data points in the principal components’ space. Other explored target Tasks (blue, purple, green and red) have centroids of varying distance to the Base Task. However, it is crucial to remember that the PCA operates on the output (i.e., the farfield scattering patterns). Therefore, close proximity in the output space (codomain) does not guarantee the same in the input space (domain). In other words, the two overlapping points from the TM and base problems may not correspond to the same input geometry features. None-the-less we find that the centroid distance of the Target task clusters in Figure 6(a) correlates relatively well with the critical transfer learning metrics presented in Table 2. As shown in Figure 6(b) we plot the PCA centroid distance and *r*_*D*_ to reach a test MARE = 0.03 (open circle symbols) and *r*_*e*_ for *D* = 1000 (open triangle symbols).

## 4 Discussion

We have demonstrated that transfer learning can be useful for solving different types of AEM problems. Specifically we showed that the knowledge gained from one AEM problem, stored in the parameters of a pre-trained DNN, can be applied to solve a different AEM problem with alternative settings. The method can be adapted to various changes in material choice, element shape, and disparate incident excitations. Although our study is only a small subset of all possible scenarios, we believe that transfer learning can be valuable for AEM problems in general. A crucial consideration for transfer learning is how dissimilar the target problem is compared to the base problem. Significant difference between base and target problems, for example such as a change in incident angle, can lead to decreased transfer learning effectiveness. Also, we found that as we increased the ground truth dataset size for the target problem, the advantage of transfer learning became less advantageous. While our five test cases showed that transfer learning is highly effective in all cases, there may be situations where it is not beneficial, or even yields worse performance if the problems being compared are too different. A positive correlation between the two critical metrics *r*_*D*_ and *r*_*e*_ and the centroid PCA distance of the far-field was found, but future research should look for such correlations in the input dimension.

Although transfer learning has been shown to always be useful between two different simulated datasets, we believe it can be applied in real-world situations as well. We propose using this method with models based on ideal simulations to deal with issues related to fabrication and measurement. This means adjusting the model to account for realistic factors using a small set of experimental data. One significant benefit of this approach is that we can predict uncertainties without needing to do a large number of individual experiments, of order of 10^3^, that are typically needed to develop an accurate deep learning model. Through use of a model transferred from simulation to experiment we can save time and resources by estimating these uncertainties, suggesting that transfer learning can be a valuable tool in both theoretical studies and practical applications of metamaterials.

## 5 Conclusions

The transferability of knowledge from a well-trained neural network to problems with different materials and external conditions has been demonstrated. The transfer learning technique presented here allows for the training of a neural network surrogate model to achieve a desired level of accuracy using substantially less data – in some cases two orders of magnitude. A key technique to enabling this approach is that we keep the dimensionality of the input and output spaces consistent among different tasks, thereby ensuring the same neural network (ResNet) architecture can be utilized for all tasks. Our study highlights a viable approach for metamaterial problems that share the same underlying physics: a neural network trained for one problem can be reused and fine-tuned for a new problem with only a small number of new data points. Transfer learning opens doors to countless captivating physical processes ripe for exploration. The sheer breadth of AEMs and associated phenomena warrant further investigation, paving the way for exciting future studies. This discovery also paves the way for dynamically updating neural network models in response to changing environments or design targets, thereby helping to mitigate the data bottleneck problem in deep learning.
